# A Double-Edged Sword: The Role of VEGF in Wound Repair and Chemoattraction of Opportunist Pathogens

**DOI:** 10.3390/ijms16047159

**Published:** 2015-03-30

**Authors:** Eric Birkenhauer, Suresh Neethirajan

**Affiliations:** BioNano Laboratory, School of Engineering, University of Guelph, Guelph, ON N1G 2W1, Canada; E-Mail: birkenhe@uoguelph.ca

**Keywords:** microfluidics, wound bacteria, *Pseudomonas aeruginosa*, biofilms, chemotaxis, vascular endothelial growth factor

## Abstract

Wound healing is a complex process essential to repairing damaged tissues and preventing infection. Skin is the first line of defense, a chief physical barrier to microbe entry. Wound healing is a physical rebuilding process, but at the same time it is an inflammatory event. In turn, molecules for wound repair are secreted by fibroblasts and others present at the wound site. Vascular endothelial growth factor (VEGF) is a critical cytokine that exhibits chemoattractant properties, recruiting other immune cells to the site. Although generally beneficial, VEGF may also act as a chemoattractant for invading microorganisms, such as *Pseudomonas aeruginosa.* *P. aeruginosa* is problematic during wound infection due to its propensity to form biofilms and exhibit heightened antimicrobial resistance. Here, we explored the influence of VEGF gradients (in a microfluidic device wound model) on the motility and chemotactic properties of *P. aeruginosa*. At lower concentrations, VEGF had little effect on motility, but as the maximal concentration within the gradient increased, *P. aeruginosa* cells exhibited directed movement along the gradient. Our data provide evidence that while beneficial, VEGF, in excess, may aid colonization by *P. aeruginosa*. This highlights the necessity for the efficient resolution of inflammation. Understanding the dynamics of wound colonization may lead to new/enhanced therapeutics to hasten recovery.

## 1. Introduction

The skin acts as a vital protective barrier that prevents microorganisms from entering into the underlying tissues and organs of the human body. Areas where the integrity of the skin is compromised by a wound can allow microorganisms ready access to the underlying tissues, leading to local and/or systemic infection if not clearly locally [1]. At the same time, wound healing is an ordered and complex process. Wound healing in healthy individuals begins within minutes of wounding, while full wound healing and tissue reconstruction can take months, even years in some cases, to be successfully completed.

The basic stages of wound healing include hemostasis, inflammation, repair, which includes cell replication and extracellular matrix (ECM) synthesis, and tissue remodeling [2]. Wound damage can also be characterized by the depth of the lesion and is classified as partial- or full-thickness wounds [3]. Partial-thickness wounds are distinguished by damage that penetrates as far as the dermal level, while full-thickness wounds involve damage that penetrates to the hypodermis and underlying muscle tissue [3]. In most cases, partial-thickness wounds will heal simply through re-epithelialization. However, this is not the case for full-thickness wounds, where a substantial portion of the dermal tissue is lost. In this case, ECM-producing cells (e.g., fibroblasts) actively proliferate while producing ECM components (primarily collagen) to fill the wound with scar tissue [3]. A reduction in epithelial thickness, compared to previously unwounded skin, is often apparent in full-thickness wounds after healing.

After the scar tissue forms in a full-thickness wound, the keratinocytes present will attempt to re-epithelialize the tissue, replacing the temporary scar tissue [2,3]. Cell migration and neovascularization in the newly formed scar tissue are promoted by the release of specific cytokines, including fibroblast growth factor 2 (FGF-2) and vascular endothelial growth factor (VEGF) [3]. The ECM tissue also acts as a scaffold and conduit for newly formed capillaries that provide nutrients to incoming and proliferating cells. VEGF is but one of many critical cytokines involved in wound healing. VEGF is predominantly secreted by keratinocytes located at the wound periphery [3]. FGF-2 and other factors, such as tumor necrosis factor α (TNF-α), are released from both macrophage and endothelial cells. Other components of the ECM, most notably hyaluronic acid, have also been shown to help promote angiogenesis and cell migration to the wound site [3]. It should be noted that neovascularization is crucial for proper wound healing; improper or incomplete neovascularization can lead to the formation of venous ulcers, resulting in a chronic/unhealed wound [3].

Regardless of severity, an infection begins with contamination, which may initially be present in the form of sparse populations of generally harmless microorganisms at the wound site [1]. When conditions are ideal for these microorganisms to begin to proliferate and adhere to the wound site, this can be described as colonization [1]. In some cases, colonization may not lead to infection. Ultimately, a critical colonization level must be reached in order for a true infection to occur [1,4]. Critical colonization refers to a specific colony-forming unit per milliliter (CFU/mL) concentration that is necessary for an infection to occur [4]. This value varies between species and is greatly influenced by the relative health and age of an individual. A study by Asada *et al.* (2012) showed that rats with full-thickness wounds infected with *Pseudomonas aeruginosa* PA01 at a concentration considered below the critical colonization actually experience accelerated wound healing rates compared to sterile wound sites [4]. This can likely be attributed to an increased immune response and subsequently a rapid activation of tissue repair systems [4]. As expected, rats whose wounds were exposed to concentrations of cells above the critical colonization level experienced significant infections and delayed wound healing.

The majority of microorganisms isolated from wound sites can be traced back to the patient’s own natural skin microbiota [5]. Colonization by these microorganisms does not tend to lead to infection under normal circumstances in healthy individuals. These microorganisms are therefore classical examples of opportunistic pathogens [5]. Common bacterial constituents of the skin microbiome include Streptococci (e.g., *Streptococcus pyogenes*), Propionibacteria (e.g., *Propionibacterium acnes*), Staphylococci (e.g., *Staphylococcus aureus*), and Pseudomonad (e.g., *P. aeruginosa*) [5]. For the purposes to this study, we elected to examine *P. aeruginosa* due to its prevalence and association with complications/resistance in wound infections.

*P. aeruginosa* is a motile Gram-negative rod-shaped bacterium with unipolar flagella [5]. It also contains type IV pili (TFP), which play important roles in both attachment and motility (e.g., twitching motility) [6,7]. The mechanisms by which TFP are used by *P. aeruginosa* to attach to and “crawl” across surfaces have been studied at length [6,7,8]. Similar to *Staphylococcus aureus* and methicillin-resistant *S. aureus* (MRSA), *P. aeruginosa* is a prolific biofilm former that can initiate biofilm production rapidly (e.g., within ten hours of inoculating plastic cover slips) [9]. It is found on the skin, but is a native soil-inhabiting microorganism with a high affinity to water [10,11,12]. Due to its high water affinity, *P. aeruginosa* tends to prefer mucosal surfaces (e.g., skin, gastrointestinal tract, oral cavity, upper respiratory tract, and distal regions of the reproductive system). As such, it is commonly found in the lungs of individuals suffering with cystic fibrosis [10,13]. It is also known to cause of dermatitis infections [5].

*P. aeruginosa* is a siderophore-producing bacterium, which makes it particularly well-adapted to life at wound sites where it can actively scavenge iron (commonly a growth limiting factor) from damaged erythrocytes [13]. In some cases, it can even share its siderophores with non-siderophore-producing microorganisms (e.g., *S. aureus*), which in turn aids the growth of other pathogenic microorganisms [13]. If iron is limited at a wound site, *P. aeruginosa* will actively lyse surrounding competitor microorganisms to obtain this essential metal, which is a cofactor for many cellular reactions [13]. Members of the Pseudomonad family are also able to rapidly acquire genes necessary for antimicrobial resistance, while sharing them amongst themselves through horizontal gene transfer mechanisms. This organism is therefore an excellent model organism for studying antimicrobial resistance gene acquisition [5,11]. Due to their ability to rapidly accept and acquire antimicrobial resistance genes, chronic infections containing *P. aeruginosa* can be difficult to treat and require an ever-evolving treatment paradigm.

Therefore, we employed a novel microfluidic system to study *P. aeruginosa* biofilm formation. We focused on the impact of VEGF concentrations on directed movement, because this is an important aspect of migrating to and colonizing a surface *in vivo* (e.g., a wound site), while VEGF is an abundant chemoattractant that is involved in wound healing and is secreted at the wound site. We ultimately found that *P. aeruginosa* cells do show an increase in directed movement towards VEGF, especially with higher concentrations of VEGF. This may have critical implications in wound infections, as VEGF, while a necessary component of the immune response, may be beneficial to opportunist pathogens when the concentrations are sufficient to attract them.

## 2. Experimental Section

### 2.1. Bacterial Strains and Cell Culturing Conditions

*P. aeruginosa* BK-76 was used throughout this study. *P. aeruginosa* BK-76 was isolated from a canine ear skin infection and obtained as a gift from the Ontario Veterinary College. Overnight cultures were grown in 6 mL tryptic soy broth (TSB; Gibco, part of ThermoFisher, Waltham, MA, USA) for 24 h at 37 °C and 200 rpm. A 100 μL volume of this culture was inoculated into 5 mL fresh TSB and incubated for 4 h to dilute the cells and allow them to reach exponential phase. Exponential phase cells were then inoculated into the microfluidic device for motility studies.

### 2.2. Microfluidic Motility Assays

The microfluidic device used to analyze *P. aeruginosa* BK-76 single-cell swimming dynamics in the presence of varying VEGF concentrations consists of a central seeding/viewing channel and two feeder channels. Feeder channels are connected to the central channel by ~800 nm pores at the viewing window that generate chemical gradients. Inlet and outlet holes are placed at the ends of the channels to allow for the entry and exit of fluids from the device. *P. aeruginosa* BK-76 cells were then inoculated into the central seeding channel where the micro-environment can be manipulated by the solutions in the feeder channels.

**Figure 1:**
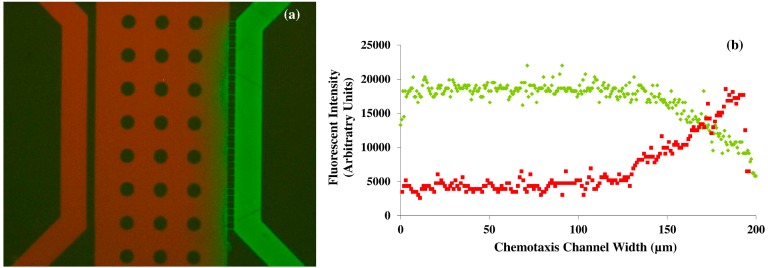
Diffusion assay microfluidic device characterization. (**a**) Representative overlaid fluorescent image showing the concentration gradient of fluorescein and Texas Red dyes; (**b**) Diagram showing the averaged pixel intensity of the dyes across the center channels width. Both dyes were flowed through the device at rates of 30 μL/h. Reproduced from [14] with permission from the Royal Society of Chemistry.

**Figure 2:**
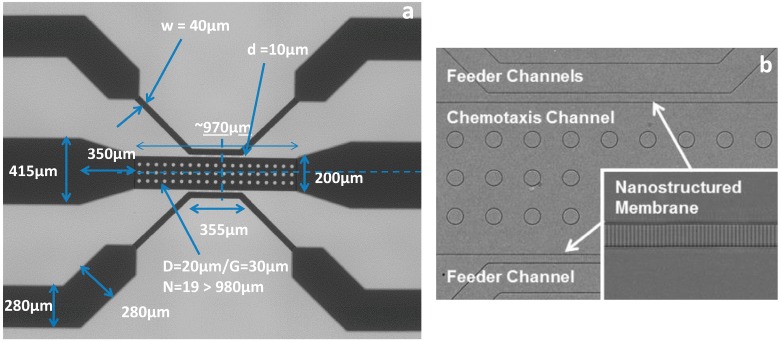
Microfluidic device dimensions. (**a**) SEM image of the microfluidic silicon device mold with channel dimensions displayed; (**b**) The main channel area (labeled chemotaxis channel) and feeder channels of the final polydimethylsiloxane (PDMS) microfluidic device as viewed using Inverted Light Microscopy (ILM). The inset image on (**b**) shows a magnified image of the feeder channel wall with connecter channels (~800 nm wide). The overall device channel depth is 10 μm. Posts in the center channel are for structural support in order to prevent channel collapse. Reproduced from [14] with permission from the Royal Society of Chemistry.

Initial device characterization consisted of a diffusion assay using fluorescein and Texas Red (both at 5 μM concentrations), which have established molecular weights (332.31 and 625.15 g/mol, respectively). Both dyes were injected into feeder channels, separately, at a rate of 30 μL/h. The diffusion of each dye was viewed using a Nikon Eclipse Ti inverted microscope, a Nikon DS-QiMc microscope camera and Nikon NIS Elements BR version 4.13 software (Nikon Instruments Inc., Melville, NY, USA) with green and red optical filters. The diffusion profile for each dye is shown in Figure 1. The microfluidic silicon device mold was further characterized under a scanning electron microscope (SEM) to acquire device dimensions (Figure 2). Figure 2 also shows an Inverted Light Microscopy (ILM) image of the final Polydimethylsiloxane (PDMS) device main channel area (labeled chemotaxis channel) and feeder channels.

### 2.3. Device Fabrication

**Figure 3:**
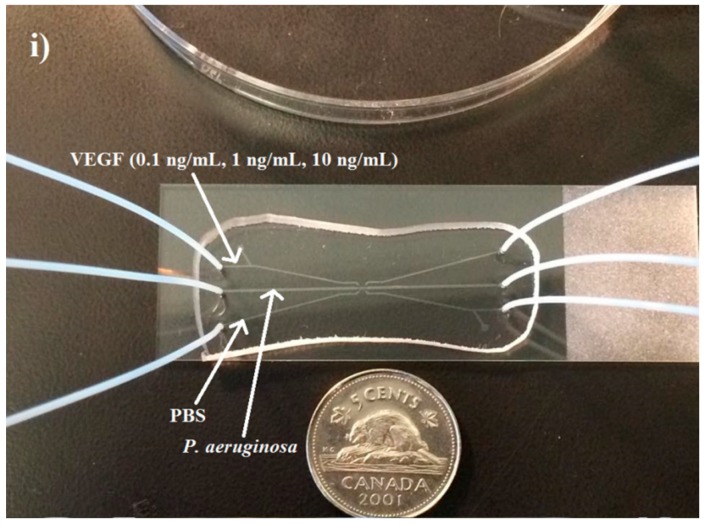

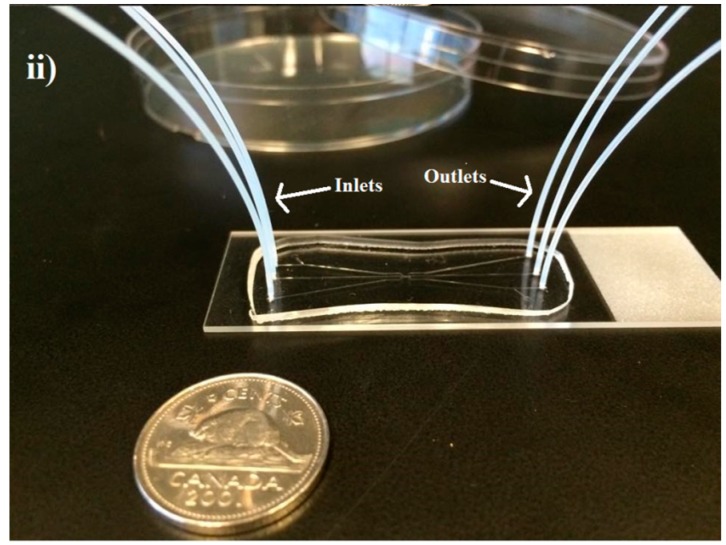
Final fabricated PDMS device bonded to a glass cover slip with inlet and outlet tubes added (**i**) and (**ii**). A Canadian five cent coin is used to show the relative scale of the completed device. (**i**) The microfluidic device as viewed from above showing the relative VEGF concentrations used; (**ii**) Side view of the device showing the inserted inlet and outlet tubing more clearly.

Microfluidic device assembly began with the preparation of the master mold via the process of silanization under a vacuum in a desiccator for 12 h. PDMS (15 g) was mixed in a 1:10 ratio with an elastomer curing agent and poured on top of the silanized master mold in a petri plate. The poured PDMS was then desiccated for one h to remove air bubbles. After this step, the device was baked at 60 °C for 4 h to allow the PDMS to crosslink and solidify. The PDMS device was then excised using a scalpel. Inlet and outlet holes were punched (0.75 mm), and the device was plasma cleaned along with a glass microscope slide (75 mm × 25 mm × 1 mm) for 30 s. The PDMS device and glass slide were placed together and allowed to permanently bond. An example of the completed microfluidic device, with inlet and outlet tubing attached, is shown in Figure 3.

### 2.4. Experimental Setup

Prior to microbial inoculation, the microfluidic device was filled with 0.1% bovine serum album (BSA) and allowed to sit for 20 min with inlet and outlet holes plugged by PDMS blocks. After this time, channels were rinsed once with distilled water. BSA was added in order to prevent microbial attachment to the glass slide, which occurred readily without this pre-treatment. *P. aeruginosa* BK-76 was then inoculated into the center channel through the inlet tubing. Once the channel was filled with *P. aeruginosa* cells, the inlet and outlet tubing was plugged with binder clips. Baseline motility was determined for *P. aeruginosa* by flowing PBS (pH = 7.4) through both feeder channels at 30 μL/h using a syringe pump. Recombinant VEGF from human was obtained from Sigma–Aldrich (V7259, 100% concentration, Sigma–Aldrich, Oakville, ON, Canada). VEGF solutions (0.1, 1, and 10 ng/mL in PBS, pH = 7) and PBS (pH = 7.4) were then flowed through the feeder channels to create VEGF diffusion gradients across the center channel. Outlet tubing for the feeder channels was connected to waste collection beakers. After 30 min of flow, 20 s videos of the center channel were captured at 614 × 512 resolutions with a 40 ms exposure, 2× analog gain, and a frame rate of 15 fps.

### 2.5. Data Analysis of Swimming Cells

Video and image analysis was carried out using the publicly available ImageJ software (http://rsb.info.nih.gov/ij/). Captured videos were then split into 10 s sections and processed individually with the background removed to exclude non-motile cells. The default threshold algorithm was then applied to the videos, and cells were tracked manually frame by frame (using a Manual Tracking Plugin). Raw data were exported to the Chemotaxis and Migration Tool (ibidi GmbH, Munchen, Germany) for analysis and motility metrics determination. Cell metrics included the Forward Migration Index (FMI), accumulated distance, Euclidean distance, velocity, center of mass, and directness. FMI (*X* and *Y*) represent the efficiency of cell migration with regards to *X* and *Y* axes; accumulated distance refers to the total distance individual cells travelled. Euclidean distance refers to the length of the line segment connecting the initial and final positions of the cell’s trajectory. Center of mass refers to the spatial average of individual cell endpoints. Directness refers to the linearity of the cell’s movement, and is calculated by comparing Euclidean distance and accumulated distances. In total, 30 cells were analyzed at baseline and for each VEGF concentration.

### 2.6. Statistical Analysis

Statistical analysis of all data groups was performed on commercially available software (R Open Source Statistical Programming). A student’s *t*-test was used to compare groups; *p* < 0.05 was considered significant.

## 3. Results and Discussion

An accurate experimental quantification of gradients of VEGF concentration *in vivo* across the extracellular space of wound tissue is not possible. Hence, computational models are the best to determine the diffusion transport of proteins such as VEGF *in vivo* in the tissue with realistic geometry and biophysical interactions of VEGF and the extracellular matrix [15,16]. The mean diffusion coefficient of Texas Red and fluorescein were calculated to be 3.6 × 10^−7^ cm^2^·s^−1^ or about 12% of the value in nanopure water, and 1.8 × 10^−6^ cm^2^·s^−1^ or 35% of the value in nanopure water respectively. Although the molecular weight of VEGF is 38.2 kDA, the diameter of the nanoporous channels (800 nm), the length of the connecting channels of the device, and the flow rate of the solutions also influences the free and rapid diffusion of molecules in to the middle channel of the designed microfluidic platform. It is estimated that up to 500 kDA macromolecules can be diffused rapidly in the nanoporous microfluidic platform observation channel with varying flow rates. Diffusion rates depend not only on the molecular size, but also on the fluidic viscosity, and temperature. In our experiments, the device characterization consisted of a diffusion assay using fluorescein and Texas Red (both at 5 μM concentrations), which have established molecular weights). The differences in the molecular weight (2 times) and the diffusion coefficient between Texas Red and fluorescein provide sufficient information for the modeling of the diffusive transport of the molecules and for real time comparison with VEGF.

The accuracy of the gradient of the solutions inside the microfluidic device is also dependent on the pressure balance in the microfluidic diffusion that exists between the PDMS and the glass surface; and the fluid viscosity; and the molecular weight of the solution. It would be not practically feasible to take samples from the top surface of the PDMS microfluidic device during the diffusion process for further obtaining chemical analytical data, as the variations in the channel height and width will result in errors in the diffusion that may propagate throughout the fluidic network. Hence, we non-invasively obtained the fluorescent intensity profiles of two different molecular weight compounds namely fluorescein and Texas Red during diffusion inside the microfluidic chamber using microscopy techniques (Figure 1). The arbitrary units of the fluorescein intensity were converted in to the concentration of the fluids inside the microfluidic device by normalizing the data. The results from these characterization experiments can be compared with the VEGF concentration to serve as the corresponding analytical data. The evolution of concentration profiles for various gradient generations by varying flow rate and the concentration of VEGF could also be obtained by mathematical transport models [17]. A generalized mathematical model would be RF = *C*_Geometry_ η *L*/*A*^2^ where RF is the flow resistance in the channel, *C*_geometry_ is the geometrical coefficient, *L* is the channel length and *A* is the cross-sectional area of the channel.

The time of perfusion of the solution inside the central observation channel of the microfluidic platform is dependent on the flow rate of the solution, molecular weight, fluid viscosity, temperature and the channel geometry. With the designed microfluidic nanoporous platform, the image and gradient profiles were recorded after 45 s of the initial perfusion of the fluorescent dyes. The flow rate of the fluid was the determining factor in establishing the stable gradient inside the center of the microfluidic channel.

VEGF concentration gradients were generated using a three-channel microfluidic device. Three concentration gradients were generated inside the device to determine the effect of this wound-relevant protein on *P. aeruginosa* BK-76 motility. Our goal was to determine if the presence of a wound-relevant cytokine could serve as chemoattractant for *P. aeruginosa* BK-76. VEGF concentrations of 0.1, 1, and 10 ng/mL were chosen because they are representative of blood, serum, and wound-site VEGF levels prior to and during wounding. VEGF concentrations of 0.1 ng/mL are representative of serum levels prior to a wounding event [18], while 1 ng/mL represents typical blood serum levels at the seventh day of wound healing, which can reach a peak by 14th day, reaching concentrations of ~2 ng/mL [18]. Higher concentrations, such as the 10 ng/mL dose, represent a concentration that would be detected in wound fluids approximately four days after wounding [19]. VEGF serum and wound-site concentrations can vary between individuals [19]. Other factors such as age, underlying medical issues, and the severity of the wound affect the relative serum and wound-site VEGF concentration [20,21].

**Figure 4:**
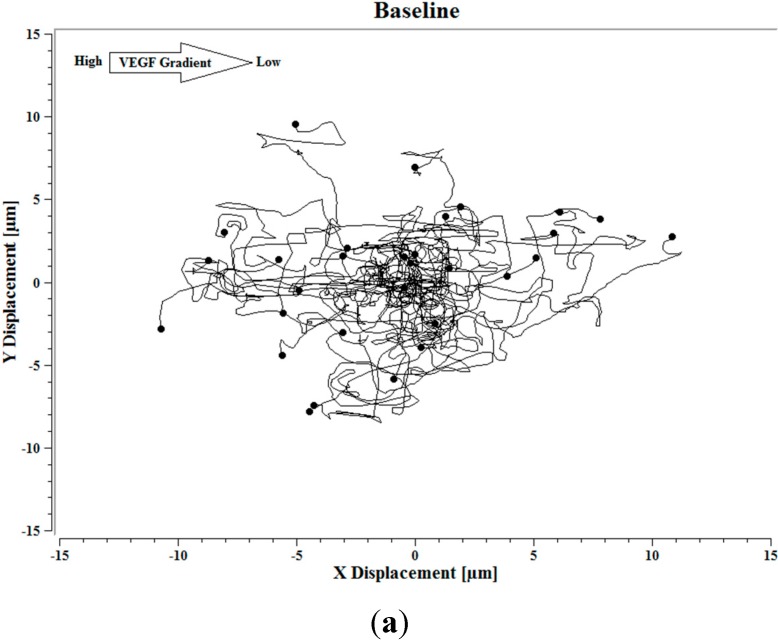

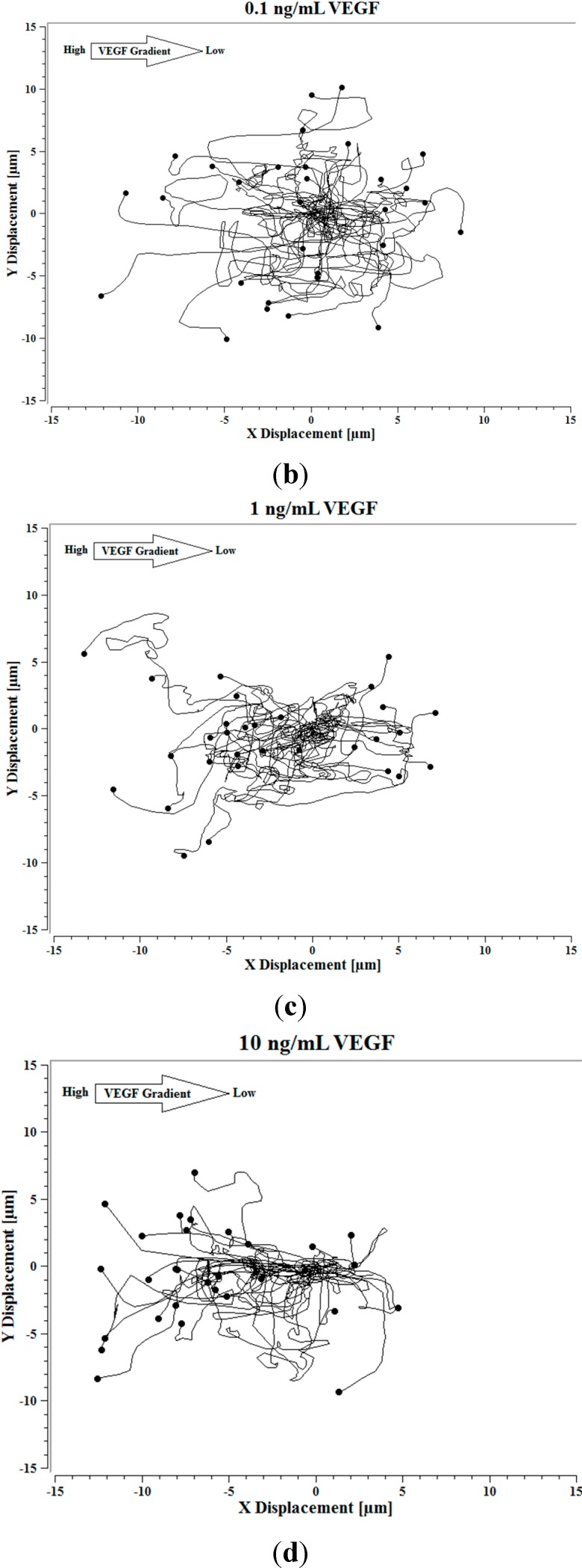
(**a**) Baseline motility and cell paths for *P. aeruginosa* BK-76 cells. Motility and cell paths of *P. aeruginosa* BK-76 cells in a 0.1 (**b**), 1 (**c**), and 10 ng/mL (**d**) vascular endothelial growth factor (VEGF) concentration gradients respectively. For context, the VEGF concentration gradient (from high to low) runs from left to right.

Image J software, with the manual tracking plugin, was used to track the movement of 30 *P. aeruginosa* cells. The motion paths of *P. aeruginosa* cells are depicted in Figure 4a–d. No noticeable differences were observed in the movement pathways between baseline and cells treated 0.1 ng/mL VEGF (Figure 4a,b). However, as the concentration of VEGF increased, changes in cell movement occurred that tracked along the concentration gradient towards the highest concentration of VEGF (Figure 4c,d).

**Figure 5:**
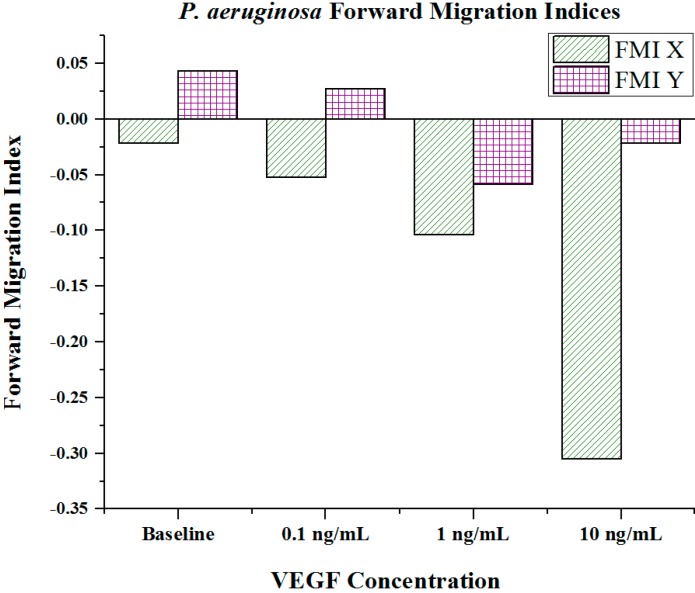
Forward migration indices of *P. aeruginosa* BK-76 cell groups in varying VEGF concentration gradients. Increasingly negative Forward Migration Index (FMI) *X* values indicate that cell groups more efficiently migrated along the VEGF concentration gradient (towards highest concentration). Negative values for FMI *X* are due to the microfluidic device orientation, as VEGF concentration gradients were generated from left (negative) to right (positive) across the chemotaxis area. Thus, migration towards the VEGF source would be shown as a negative value. (*N* = 30).

**Figure 6:**
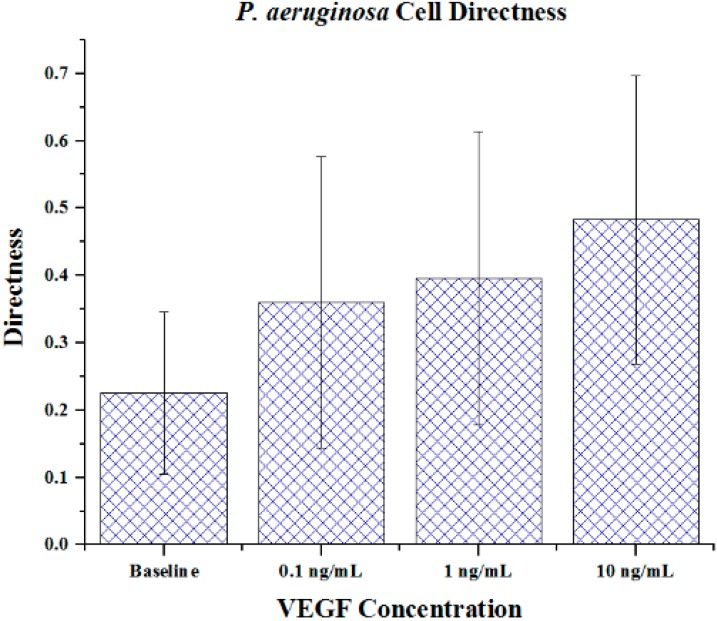
The directness of *P. aeruginosa* BK-76 cells in VEGF concentration gradients. Increasing FMI X values indicate that cell groups had more direct paths with regards to migrating along the VEGF concentration gradient (towards highest concentration). Error bars show standard deviation.

**Figure 7:**
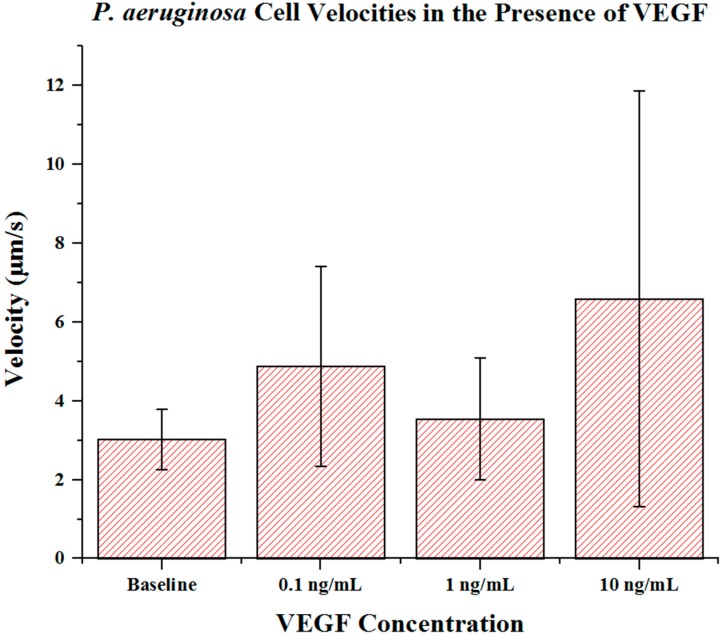
*P. aeruginosa* BK-76 cell velocities in VEGF concentration gradients. Results show that up until 10 ng/mL concentrations, *P. aeruginosa* cell velocities remain relatively similar. The highest cell speeds were seen in 10 ng/mL VEGF gradients, with the highest recorded cell speed being 26.63 μm/s. Error bars show standard deviation.

Cell metrics showed changes in the cell forward mobility index (FMI) (Figure 5), directness (Figure 6), and velocity (Figure 7) that occurs with increasing VEGF concentration gradients. The FMIs increases with increase in the VEGF concentration gradient, showing that the overall cell group’s migration efficiency towards the VEGF source is enhanced. This is demonstrated by increasingly negative FMI *X*-axis values (due to the VEGF source orientation). FMI *Y*-axis values are shown in Figure 5. The directness of individual cells increased as the VEGF concentration increased, showing cell directionality. This means that cells travelled from their initial to final positions in a more direct fashion as the VEGF concentration gradient increased; *P. aeruginosa* cells tumbled less and showed a clear attraction to VEGF sources. With regards to significance, cell directness under all VEGF concentrations was significantly different (*p* < 0.05 in all cases) than baseline values. When comparing between VEGF concentrations, we found that 0.1 and 10 ng/mL concentrations exhibited significantly different directness values (*p* = 0.023). Cell velocities did not show a well-defined overall trend (compared to FMI and directness) in regards to VEGF concentration. Velocities varied among the baseline, the 0.1 and the 1 ng/mL VEGF concentrations. The highest cell velocities were observed for cells in a 10 ng/mL concentration gradient (maximum cell velocity = 26.63 μm/s). Significant differences in velocity occurred between baseline, 0.1 and 10 ng/mL concentrations (*p* < 0.001 in both cases). When comparing among VEGF concentrations, significant differences were observed between 0.1 and 1 ng/mL (*p* = 0.018) and between 1 and 10 ng/mL concentrations (*p* < 0.005).

Extraneous cell metrics, such as center of mass, accumulated distance, and Euclidean distance are summarized in Table 1. From these measures, we noted that the center of mass the *X* component increased (in magnitude) with increasing VEGF concentrations. This again shows that the center of the observed cell groups shifted towards the VEGF stimulus, particularly as the concentration increased. This shift was naturally more apparent under higher VEGF concentrations. Decreasing accumulated distances and increasing Euclidean distances were paired with increasing VEGF concentration gradients, showing that the cells moved with increased directionality in the presence of higher VEGF concentrations. This was expected, as the accumulated distance and Euclidean distance values are used for directness calculations.

Findings of the unpublished experiments from the BioNano Laboratory shows evidence that the dynamic range and transient presence of specific amino acids such as arginine and glutamine, at particular stages during wound infection may heavily influence the motility of *Pseudomonas aeruginosa.P. aeruginosa* has the ability to positively and negatively respond to a variety of amino acids including arginine and glutamine at various concentrations [22]. For example, *Pseudomonas strain* RW1 was attracted to amino acids such as glycine, histidine, leucine, proline and tryptophan while *Pseudomonas fluorescens* was not [23].

**Table 1:** Extraneous *P. aeruginosa* BK-76 cell swimming metrics. Center of mass describes the overall change in position of the cell group’s center. Increasingly negative *X* values indicate that cell group has more efficiently migrated along the VEGF concentration gradient (towards highest concentration). Negative values for the center of mass *X* are due to the microfluidic device orientation, as the VEGF concentration gradients were generated from left (negative) to right (positive) across the chemotaxis area. Thus, a cell group’s shift towards the VEGF source would be shown as a negative value. Accumulated and Euclidean distances are used in determining the directness of cell motility. Accumulated distance represents the total distance travelled. Euclidean distance represents the distance between the start and end point of a cell’s trajectory.

Characteristics	Baseline	0.1 ng/mL	1 ng/mL	10 ng/mL
Center of Mass *X*	−0.98	−0.69	−2.39	−5.87
Center of Mass *Y*	0.52	−0.08	−0.81	−0.75
Accumulated Distance (μm)	26.30 ± 8.63	23.78 ± 12.72	19.02 ± 9.35	17.83 ± 9.49
Euclidean Distance (μm)	5.68 ± 3.03	6.89 ± 2.86	6.34 ± 3.15	7.61 ± 3.76

## 4. Conclusions

VEGF is a crucial proangiogenic cytokine that is upregulated in response to wounding and bacterial infections. It is responsible for promoting neovascularization at the wound site. We initially hypothesized that VEGF would act as a chemoattractant and that increasing VEGF concentration gradients would lead to biases in cell movements, especially as the concentration of VEGF increased across the gradient. While our hypotheses were confirmed, we intend to investigate other cytokine and wound-related molecules and their impact on the motility of *P. aeruginosa* in the future. This study suggests that VEGF concentrations at and around the perimeter of wound sites (typically ≥ 10 ng/mL) act to attract possible motile opportunistic pathogens to the wound site. Other elements of wound healing obviously combat the minor effects that VEGF may have as a chemoattractant.
